# A Veterinary Vaccine for SARS-CoV-2: The First COVID-19 Vaccine for Animals

**DOI:** 10.3390/vaccines9060631

**Published:** 2021-06-10

**Authors:** Vivek P Chavda, Jack Feehan, Vasso Apostolopoulos

**Affiliations:** 1 Department of Pharmaceutics and Pharmaceutical Technology, L M College of Pharmacy, Ahmedabad-380008, Gujarat, India; vivek.chavda@lmcp.ac.in; 2 Institute for Health and Sport, Victoria University, Melbourne, VIC 3030, Australia; jack.feehan@vu.edu.au

As coronaviruses have a wide range of host species, many animals harbor these pathogens, however only a handful of them become severely infected. It has been confirmed that the spread of the SARS-CoV-2 virus has a zoonotic link, i.e., transmission between human and animal [1]. According to the World Health Organization, vaccines may protect infected animal species and prevent transmission of viral mutations. In 2020, Denmark culled 17 million mink as a study suggested that infected animals had a viral mutation that could be transmitted to humans [2]. In the US and other countries, there have been pet animals such as cats and dogs registered as being infected with SARS-CoV-2. Some zoo animals have also been found positive for SARS-CoV-2 after showing symptoms of infection [3]. These infected animals may become ill or instead show no symptoms. Severe disease in animals appears to be uncommon.

To combat the threat of animal-to-human transmission, and the rise of mutant variants, Russian researchers from the Federal Service for Veterinary and Phytosanitary Surveillance have developed the first COVID-19 vaccine (Carnivac-Cov) for animals. The vaccine, also known as Karnivak-Kov, has been designed for carnivores (Figure 1). The department has stated that research trials on arctic foxes, cats, rats, mink, and other species were completed in October 2020 and that the data were satisfactory in terms of safety and efficacy. Based on the data generated, SARS-CoV-2 immunity was reported to last at least for six months after the vaccination [4]. Based on the reported clinical trial findings, it was suggested that Carnivac-Cov was safe and was capable of producing immunity in all animals enrolled in the study. Carnivac-Cov is based on the inactivated vaccine development platform. SARS-CoV-2 is first inactivated through chemical means, heat, or radiation. This kind of treatment makes the virus replication-deficient; however, it can be recognized by the host immune system to elicit humoral and T cell-mediated immune responses to the viral antigens [5]. This vaccine for animals was created partly as a public health intervention, in the case of the virus spreading from animals to humans or, in a worst-case scenario, mutating in animals and then spreading back to humans in a more virulent form. It could also provide a strategy to aid animal industries, some of which have been ravaged by pathogens in recent times [6]. This vaccine was initially studied using ferrets as animal testing species that were less vulnerable to SARS-CoV-2, and after promising effects, tests on susceptible animals began. Russian fur farms as well as companies in Greece, Poland, and Austria have decided to purchase and roll out the vaccine [7]. The vaccine will not only protect the infected animals but may also aid in the prevention of the transmission of viral mutations. According to the Russian regulator, existing manufacturing potential is 3 million doses every month, ideally increasing to 5 million in the near future. The first batch of 17,000 doses has already been manufactured and delivered in Russia.

While the global focus is on the vaccine roll-out in humans, SARS-CoV-2 infections in animals could be a vital part of pandemic recovery, as well as in future preparedness. While the results of the trials are yet to be published or released, the early reports are promising. While scrutiny of the data will be important, a successful animal vaccine could be very impactful in the future.

## Figures and Tables

**Figure 1:**
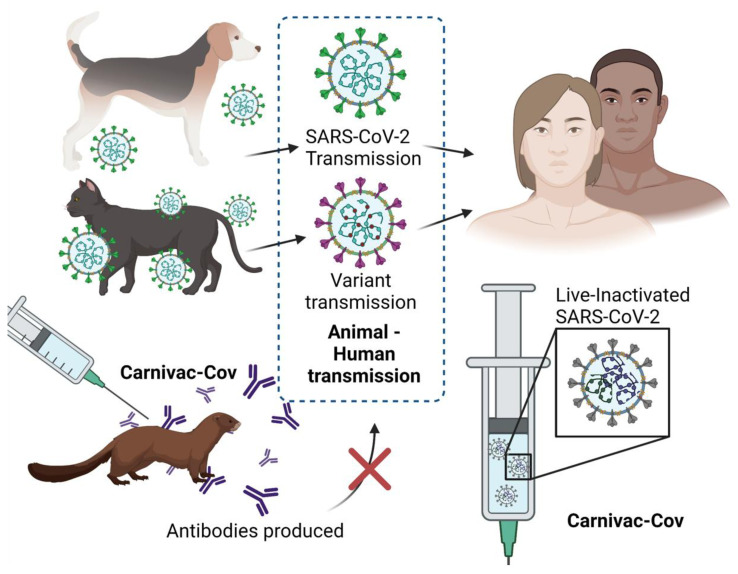
Schematic illustration of the live-inactivated SARS-CoV-2 vaccine Carnivac-Cov, which induces immune responses in animals and could potentially prevent against animal-to-human transmission. The figure was created with biorender.com.
